# Optimal Dose Levels in Screening Chest CT for Unimpaired Detection and Volumetry of Lung Nodules, with and without Computer Assisted Detection at Minimal Patient Radiation

**DOI:** 10.1371/journal.pone.0082919

**Published:** 2013-12-26

**Authors:** Andreas Christe, Zsolt Szucs-Farkas, Adrian Huber, Philipp Steiger, Lars Leidolt, Justus E. Roos, Johannes Heverhagen, Lukas Ebner

**Affiliations:** 1 Department of Radiology, Hospital and University of Bern, Inselspital, Bern, Switzerland; 2 Department of Radiology, Hospital Center of Biel, Biel, Switzerland; 3 Department of Radiology, Duke University, Durham, North Carolina, United States of America; University of Groningen, The Netherlands

## Abstract

**Objectives:**

The aim of this phantom study was to minimize the radiation dose by finding the best combination of low tube current and low voltage that would result in accurate volume measurements when compared to standard CT imaging without significantly decreasing the sensitivity of detecting lung nodules both with and without the assistance of CAD.

**Methods:**

An anthropomorphic chest phantom containing artificial solid and ground glass nodules (GGNs, 5–12 mm) was examined with a 64-row multi-detector CT scanner with three tube currents of 100, 50 and 25 mAs in combination with three tube voltages of 120, 100 and 80 kVp. This resulted in eight different protocols that were then compared to standard CT sensitivity (100 mAs/120 kVp). For each protocol, at least 127 different nodules were scanned in 21–25 phantoms. The nodules were analyzed in two separate sessions by three independent, blinded radiologists and computer-aided detection (CAD) software.

**Results:**

The mean sensitivity of the radiologists for identifying solid lung nodules on a standard CT was 89.7%±4.9%. The sensitivity was not significantly impaired when the tube and current voltage were lowered at the same time, except at the lowest exposure level of 25 mAs/80 kVp [80.6%±4.3% (p = 0.031)]. Compared to the standard CT, the sensitivity for detecting GGNs was significantly lower at all dose levels when the voltage was 80 kVp; this result was independent of the tube current. The CAD significantly increased the radiologists’ sensitivity for detecting solid nodules at all dose levels (5–11%). No significant volume measurement errors (VMEs) were documented for the radiologists or the CAD software at any dose level.

**Conclusions:**

Our results suggest a CT protocol with 25 mAs and 100 kVp is optimal for detecting solid and ground glass nodules in lung cancer screening. The use of CAD software is highly recommended at all dose levels.

## Introduction

Image noise in computed tomography (CT) is mainly caused by photon statistics, also known as quantum noise. Photon starvation and electronic noise become significant at low dose levels and with obese patients. Tube current and voltage are two important user-selectable parameters in CT imaging that can influence quantum noise. With lower tube currents or voltage it is possible to reduce the applied radiation dose to patients; however, this reduction will also increase the quantum noise and will decrease the image quality [1].

In the past, several studies have shown that it is possible to lower the tube current, and therefore also lower the radiation dose, in CT imaging without a major loss of objective or subjective image quality [2]–[13]. To maintain diagnostic quality in the detection of lung nodules, tube currents can be reduced to well below 100 mAs at a constant voltage of 120 kVp [2], [3], [11]. Some authors proposed reducing the current to 80, 70, 60 or even 10 mAs [4]–[12], which corresponds to dose reductions of 50–84%. Some studies even defined threshold tube currents of 20 mAs to detect ground glass nodules (GGNs), alveolar consolidations and lung nodules [3], [13]. Another approach to reducing the radiation dose in CT imaging is to decrease the tube voltage. High voltages from 100–140 kVp are still widely used; however, promising results suggest that a voltage reduction to 80 kVp is possible [14]–[17]. There is very little data on the combination of reduced kVp and mAs for lung nodule detection. Phantom studies are well suited for detecting the threshold levels at which diagnostic accuracy is not altered and the patient is not exposed to accessory radiation.

A low-dose protocol would not only protect the population undergoing screening CT imaging for early lung cancer detection but would also reduce the cumulative radiation dose of follow-up CT exams in patients with suspected lung metastases, incidental lung nodules, tuberculosis and pulmonary fungal infections.

The usefulness of computer-aided detection (CAD) software for lung nodule detection has been published previously [19]–[23]. However, Lee et al. [23] showed that the sensitivity of a CAD system (81%) did not significantly differ from the sensitivity of radiologists (85%). Radiologists were better able to detect nodules attached to other structures, whereas the CAD was better at detecting isolated nodules and nodules that were ≤5 mm in diameter [23].

Recently published articles have indicated that the combination of radiologists and CAD could increase detectability between 2 and 14% [18]–[20]. Hein et al. described the feasibility of low-dose CAD at 5 mAs and 120 kVp [24]; however, there have been few reports on the accuracy of using low-dose CAD as a standalone tool or in combination with radiologists.

Low-dose CT imaging has been criticized for its lack of accuracy in nodule size measurement, which is critical for follow-up examinations. One study showed no significant impact on volume measurement with the CT dose [2], while another group found that nodule volume was underestimated at lower doses [25]. The aim of this phantom study was to minimize the radiation dose by finding the best combination of low tube current and low voltage that would result in accurate volume measurements when compared to standard CT imaging without significantly decreasing the sensitivity of detecting lung nodules both with and without the assistance of CAD.

## Materials and Methods

### Lung Phantom

**Figure 1:**
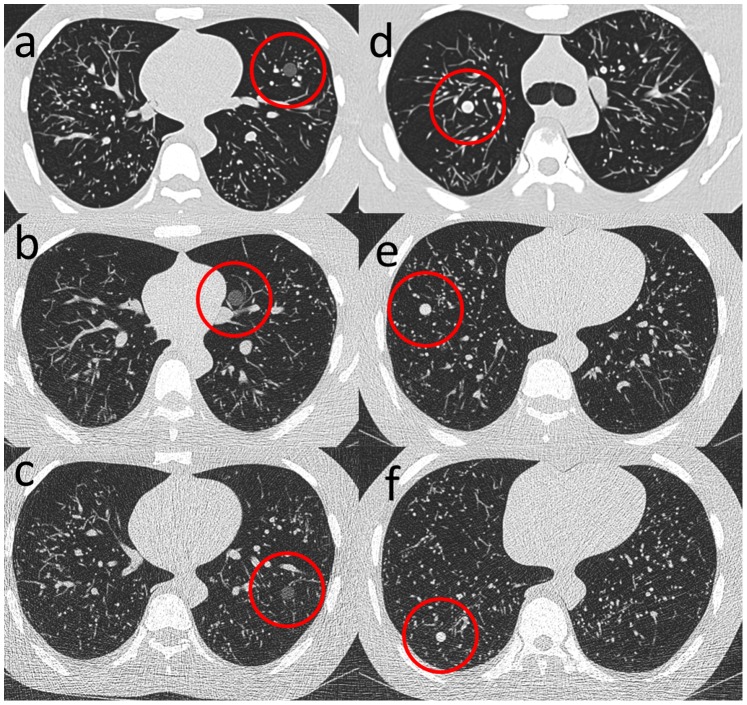
Anthropomorphic chest phantom with artificial lung nodules at different dose levels. At the standard CT dose level of 100/120 kVp (**a**), the 10-mm ground glass nodule (GGN) is easily detectable. At the lowest exposure level of 25 mAs/80 kVp (**c**), the 10-mm GGN is still visible, but the sensitivity is significantly impaired. The lowest acceptable dose level without a loss of sensitivity for detecting GGNs (12 mm) was 25 mAs/100 kVp (**b**). For solid nodules, the decrease in detectability was less obvious from the standard dose with a 10-mm nodule (**d**) to the lowest dose with an 8-mm nodule (**f**). However, for maintaining sensitivity, a dose of 25 mAs/100 kVp is necessary. (**e**), Example of a 10-mm nodule. The phantom received only 22% of the standard CT dose (**a, d**) at the lowest acceptable exposure level (**b, e**).

A commercially available lung phantom (Chest Phantom N1, Kyoto Kagaku Co., Ltd, Kyoto, Japan) was used (Fig. 1). This anthropomorphic phantom is an accurate life-size anatomical model of a male human torso with synthetic heart, trachea, pulmonary vessels (right and left) and abdomen (diaphragm) block. The thickness of the chest wall is based on clinical data measurements. The x-ray absorption rates of the soft tissue substitute material (polyurethane, gravity 1.06) and synthetic bones (epoxy resin) are very similar to human tissue absorption rates. The arms-abducted torso position was appropriate for CT scanning. The pulmonary vessels are spatially traceable.

The phantom size was 43 cm × 40 cm × 48 cm (height), which is based on an average Japanese male with a body weight of 70 Kg.

### Image Acquisition

CT images of the chest phantom were obtained with a 64-row multi-detector CT scanner Somatom Sensation 64 (24×1.2 mm, pitch 0.8, slice thickness 1.5 mm, increment 1.5 mm by Siemens, Forchheim, Germany). The scan length and field of view were kept identical at 35 cm and 33 cm, respectively. Three tube currents of 100, 50 and 25 reference mAs were combined with three tube voltages of 120, 100 and 80 kVp, which resulted in nine different protocols (reference mAs/peak voltage): 100/120, 100/100, 100/80, 50/120, 50/100, 50/80, 25/120, 25/100 and 25 mAs/80 kVp. The 100 ref mAs/120 kVp combination represents the standard image quality that is used routinely in Europe [26], [27]. The other eight kV/mAs combinations are referred to as low-dose protocols. Automatic current modulation (CareDose4D) was used to approximate the routine scans and to maintain independence from body weight and body mass index (BMI). Based on the image quality reference mAs, the scanner was able to adapt the tube current for each scan position (effective mAs) and to obtain the same specific image quality (reference mAs) over the entire scan length based on the size of the patient. Images from the Somatom Sensation were reconstructed using the classic filtered back projection with a lung kernel of B60 for best diagnostic accuracy and to meet the preset CAD. Dose was represented by the dose length product DLP (mGycm) provided by the scanner.

### Artificial Nodules

Four artificial solid nodules (+100 Hounsfield units [HU]; 5, 8, 10 and 12 mm) and 4 artificial GGNs (−630 HU; 5, 8, 10 and 12 mm) from KYOTO KAGAKU® (Kyoto, Japan) were used (Fig. 1). To simulate a realistic cancer patient, 0 to 8 nodules were placed in all lung segments; the number, location and size of the nodules were chosen at random. To detect a significant drop (5%) in sensitivity (estimate of 85% at the standard-dose CT) with a statistical power of 80% at an alpha level of 0.05, 62 nodules per kV/mAs combination were needed. To prevent recognition bias, nodules were rearranged for each dose level: the phantom was bilaterally filled with a random number of solid nodules and GGNs until the number of nodules in both categories reached at least 62. This process was repeated for each dose level. The lung segment, side and size were also randomly assigned to each nodule; therefore, the average nodule size should be the mean of the four sizes (8.75 mm). The nodules were placed between 1 and 5 cm away from the chest wall to ensure the same diagnostic difficulty. On average, 19 phantoms were needed. In addition, 4 normal phantom scans were added at each dose level. In total, there were 127 to 143 solid nodules and GGNs at each dose level, with a sum of 1209 nodules (579 solid and 630 GGN) for the nine protocols.

### Image Analysis

Three blinded radiologists with 5, 3 and 2 years of experience in thoracic radiology separately read all CT datasets on a PACS workstation (Picture Archiving and Communication System R11.4.1, 2009; Philips, Best, Netherlands; Sectra, Linkoping, Sweden). The radiologists were not part of the research group. Each nodule was registered as “yes” or “no” by capturing the nodule slice position, side and density (ground glass or solid). Radiological workstations are not yet available at all institutions worldwide for fast image reconstructions like maximum intensity projection (MIP) or multiplanar reconstruction (MPR). Therefore, radiologists were not allowed to use MIP or MPR for this study and read the axial slices only. In addition, all radiologists scored the subjective image quality on a linear scale from 1 to 5 in the lung window (level −500 HU, width 1500 HU) with hard Kernel B60f. The subjective grading scale was as follows: 1 - non-diagnostic image quality (0–20% subjective image quality); 2 - poor, diagnostic confidence significantly reduced (21–40%); 3 - moderate, but sufficient for diagnosis (41–60%); 4 - good (61–80%); and 5– excellent (81–100%). Half-point classifications were accepted. In a second reading session, all datasets were analyzed by the CAD software as a standalone reader. CT images were sent to a Median workstation (Median Technologies, Valbonne, France), where the CAD software Lesion Management Solutions LMS Version 6.0 (Median Technologies, Valbonne, France) searched for the different nodules on 1.5-mm slices. According to the manufacturer, the detection performance of LMS-CAD is optimized for solid pulmonary nodules but can also detect GGNs. A fourth reader with 2 years of experience in thoracic radiology analyzed the performance of the CAD by tracking the true-positive and false-positive findings using an answer key that detailed the nodule positions.

### Reproducibility of Volume Measurements at Lower Dose Levels

The impact of dose and attenuation on the measured size of solid nodules was analyzed in all nodules at nine dose levels (n = 579). The frequencies of the four nodule sizes of 5, 8, 10 and 12 mm should be the same after random assignment, with an average nodule size of 8.75 mm. One reader (with 5 years of experience in chest radiology) measured the longest axial diameter of all nodules digitally (according to the Response Evaluation Criteria In Solid Tumors; RECIST [28], [29]) and calculated their volumes by multiplying the longest axial axis, the perpendicular short axial axis, the longest coronal z-axis and π/6 at all nine dose levels. In a second round, the nodules were measured by the Median workstation. The computer used segmentation and threshold methods for automated volume and longest axial diameter measurement. A fourth radiologist with 2 years of experience in chest radiology performed this semi-automatic process by clicking on the designated nodule. In addition, as a standard of reference, the spherical volume was calculated by measuring the real diameters in three dimensions with a micrometer (π*d1*d2*d3)/6. With this process, absolute and relative measurement errors could be stratified for the different lesion sizes as relative error increases with a decrease in lesion size.

### Statistics

Data from the three independent human readers were averaged, and the mean and individual data were used for statistical analysis. The practicability of data pooling was tested: for each reader at each dose level the chi square test was applied to the different nodule sizes (3 degrees of freedom) with p values ranging from 0.18 to 0.77 indicating the validity of pooling the different nodule sizes (null hypothesis of data homogeneity cannot be rejected in favor of heterogeneity). A logistic regression proved the feasibility of pooling the solid and the ground glass nodules together with a p-value of 0.6 for nodule type (accepting homogeneity). The sensitivities of all ground glass and solid nodules were calculated at all dose levels. The known number of nodules per scan was used as the standard of reference. In addition, an analysis per nodule diameter and nodule type (solid or GGN) was performed separately with all nodules on all dose levels. Paired comparisons of the sensitivity at a specific dose level against the normal dose dataset were performed for each nodule size and nodule type. The null hypothesis of equality of sensitivity for each dose pair was tested by applying the Z-test of proportions (Chi-Square) [30]. The change in sensitivity for the combined sensitivity (radiologist and CAD) compared to the sensitivity of the radiologist alone was analyzed using McNemar’s test [31]. For the combined sensitivity, a positive finding was defined as either the radiologist, the CAD or both detected the nodule. To calculate the inter-observer agreement, the detection of all true-positive nodules and the classification into ground glass and solid nodules was taken into account separately for each dose level. Radiologists were compared among each other, and the agreement of each radiologist and the CAD was calculated. Mean agreements for the radiologists and the radiologists against CAD were determined. Inter-observer comparison was performed by calculating agreement levels using Fleiss’ Kappa statistics [32], [33]. The Kappa strength of agreement was as follows: <0.20 poor agreement, 0.21–0.40 fair, 0.41–0.60 moderate, 0.61–0.80 good and 0.81–1.00 very good [34]. Inter-observer agreement between ‘radiologist-radiologist’ and ‘radiologist-CAD’ was tested using the comparison of correlation coefficients [30].

Confidence intervals were used to determine agreements of volume measurements between known nodule sizes and the displayed nodule sizes on standard and on all low-dose CTs (for radiologist and the CAD measurements, respectively). The confidence interval (CI) for the measurement difference of the volume and the diameter gives the range of 95% of all volume measurement errors (VMEs) and is calculated from the standard deviation of the measurement differences [31]. To detect significant differences in the volume measurements, the CI of the VME should not include 0 for a significant systematic bias. With an increase in nodule volume at a hypothetical nodule follow-up of over the upper CI, the probability of real growth would be 95%. The standard deviation of the diameter and volume measurements of the nodules on the highest and lowest dose CT was compared using the F-test to determine the measurement variability. The Z-test was used to compare subjective image quality. The Z-test, McNemar test, F-test, Fleiss’s κ statistics and the power analysis were performed using MedCalc® Version 7.6.0.0 (MedCalc Software, Mariakerke, Belgium).

## Results

Between 127 and 143 solid nodules and GGNs were scanned at each of the nine dose levels. In total, 1209 nodules (579 solid nodules and 630 GGNs) were scanned. The average radiation exposure at each dose level is given as the dose length product in Table 1.

**Table 1:** Sensitivities of readers and CAD alone and combined for each exposure level.

							Mean sensitivity of readers alone ± standard deviation				Sensitivity CAD					
Tube parameters						DLP	tot		solid	ggn	tot		solid		ggn	
						(mGycm)										
**100 mAs**			**120 kVp**			**183.8**	**92.3%±4.1%**		**89.7%±4.9%**	**94.9%±4.4%**	**74.9%**		**91.4%**		**59.1%**	
100 mAs			100 kVp			128.4	90.7%±8.8%		89.0%±7.9%	92.3%±8.3%	75.4%		91.2%		60.2%	
100 mAs			80 kVp			71	85.9%±4.9%*		84.6%±3.3%	87.2%±9.7%*	74.0%		89.7%		58.9%	
50 mAs			120 kVp			122.5	91.1%±5.6%		88.4%±6.1%	93.5%±6.7%	60.5%*		93.3%		35.6%*	
50 mAs			100 kVp			82.6	90.0%±5.0%		89.9%±7.6%	90.0%±2.7%	60.0%*		90.3%		37.3%*	
50 mAs			80 kVp			45.9	86.9%±3.5%*		87.4%±5.7%	86.6%±4.6%*	55.3%*		82.2%		35.4%*	
25 mAs			120 kVp			61.2	90.4%±7.2%		88.2%±8.3%	92.5%±6.2%	54.1%*		88.1%		21.6%*	
25 mAs			100 kVp			40.7	88.2%±5.0%		86.6%±5.2%	89.8%±5.4%	61.6%*		86.1%		38.2%*	
25 mAs			80 kVp			22.3	**82.9%**±3.7%*		80.6%±4.3%*	85.0%±3.1%*	48.9%*		78.6%		20.6%*	
							**Mean sensitivity of readers+CAD combined ± standard deviation**				**CAD vs Radiologists**					
							**tot**		**solid**	**ggn**	**tot**		**solid**		**ggn**	
**100 mAs**	**120 kVp**						**94.8%±2.4%**		**94.8%±2.3%**	**94.9%±4.4%**	**74.9%** **		**91.4%**		**59.1%** **	
100 mAs	100 kVp						95.1%±5.5%		93.6%±5.4%	96.5%±5.8%	75.4%**		91.2%		60.2%**	
100 mAs	80 kVp						91.7%±2.8%		93.3%±2.8%	90.2%±7.7%	74.0%**		89.7%		58.9%**	
50 mAs	120 kVp						94.2%±5.1%		95.1%±3.2%	93.5%±6.7%	60.5%**		93.3%		35.6%**	
50 mAs	100 kVp						93.7%±0.8%		96.6%±1.3%	91.3%±0.8%	60.0%**		90.3%		37.3%**	
50 mAs	80 kVp						91.4%±1.7%		96.3%±2.0%	**87.2%**±3.6%	55.3%**		82.2%		35.4%**	
25 mAs	120 kVp						94.8%±2.9%		96.1%±1.3%	93.5%±4.7%	54.1%**		88.1%		21.6%**	
25 mAs	100 kVp						94.1%±3.8%		97.7%±2.5%	90.7%±5.1%	61.6%**		86.1%		38.2%**	
25 mAs	80 kVp						86.8%±3.0%*		88.6%±2.9%*	85.0%±3.1%*	48.9%**		78.6%		20.6%**	
							**Delta sensitivity of readers+CAD ± standard deviation**									
							**tot**	**solid**		**ggn**						
**100 mAs**		**120 kVp**					**2.5%±1.7%** *	**5.1%±3.6%** *		**0.0%±0.0%**						
100 mAs		100 kVp					4.4%±3.4%*	4.6%±2.5%*		4.2%±4.6%						
100 mAs		80 kVp					5.8%±2.1%*	8.7%±3.2%*		3.0%±2.6%						
50 mAs		120 kVp					3.1%±2.1%*	6.7%±4.4%*		0.0%±0.0%						
50 mAs		100 kVp					3.8%±4.2%*	6.7%±6.7%*		1.3%±2.2%						
50 mAs		80 kVp					4.4%±2.1%**	8.9%±3.8%*		0.6%±1.1%						
25 mAs		120 kVp					4.4%±4.6%**	7.9%±7.7%*		1.0%±1.6%						
25 mAs		100 kVp					5.9%±1.2%**	11.1%±2.7%**		1.0%±1.6%						
25 mAs		80 kVp					3.9%±0.7%*	7.9%±1.4%*		0.0%±0.0%						

**Note** - DLP, dose length product, tot, total nodules (solid+ggn); solid, solid nodule; ggn, gound glass nodule; mAs, Miliampèresecond; kVp, Kilovolt peek; CAD, computer assisted detection.

significant: p-value<0.05; compared to standard dose CT, to radiologists without CAD (delta) or CAD compared to radiologists (CAD vs Radiologists).

significant: p-value<0.001; compared to standard dose CT, to radiologists without CAD (delta) or CAD compared to radiologists (CAD vs Radiologists).

### Nodule Detection by Human Observers Only

The average sensitivity for detecting solid nodules on a standard CT was 89.7%±4.9 (±standard deviation [SD]) and only dropped significantly at the lowest dose level (25 mAs/80 kVp; sensitivity, 80.6%±4.3 [p = 0.031]). The sensitivity for the individual readers at each dose level is given in Appendix S1. The mean sensitivity of detecting GGNs on a standard CT was 94.9%%±4.4 and dropped significantly at 80 kVp to 87.2%, 86.6% and 85.0% for 100, 50 and 25 mAs, respectively (p from 0.02 to 0.04). Detectability was not impaired for dose levels that included 120 or 100 kVp (Fig. 1). The sensitivity for all nodules (solid nodules and GGNs together) was 92.3%%±4.1%, which also dropped significantly at 80 kVp (Table 1).

### Nodule Detection with CAD Only

At the standard dose level, CAD identified 91.4% (95% CI: 81.1 to 96.6%) of all solid nodules and only 59.1% (95% CI: 46.5 to 70.7%) of all GGNs. The sensitivity for solid nodule detection dropped to 78.6% (95% CI: 65.9 to 87.6%) at the lowest dose level, but this decrease was not significant. The capacity to detect GGNs was already impaired at 50 mAs and dropped to 20.6% (CI: 12.0 to 32.8%) at the lowest dose. Therefore, the total sensitivity of detecting all solid nodules and GGNs was also insufficient at 50 and 25 mAs with any kVp (Table 1). The average false-positive rate for one phantom scan was 13.7 on a standard CT and dropped to 4.9 at the lowest dose level.

### Human Observers versus CAD Software

At the standard dose level, the radiologists were significantly better than the CAD at detecting GGNs (94.9% and 59.1%, respectively) (p<0.0001), whereas the CAD demonstrated a non-significant higher average sensitivity for detecting solid nodules (91.4%) compared to the radiologists (89.7%) (p = 0.087). Moreover, two of the radiologists had slightly better sensitivities than the CAD, and one had a lower sensitivity (Appendix S1, p between 0.32 and 0.85). The results for the other dose levels are shown in Table 1.

### Inter-observer Variability

On average, the inter-observer agreement among the three radiologists was 0.90±0.08 (standard error [SE]) for standard CT and only dropped significantly at the lowest dose level. The K strength of agreement fell significantly to 0.80±0.09 (p = 0.0029). The agreement between CAD and the radiologists was lower: for the standard CT, the mean agreement was 0.69±0.11 (p<0.0001), whereas the mean agreement for the lowest dose was 0.52±0.13 (p<0.0001). The inter-dose variabilities for CAD and radiologists are listed in Table 2.

**Table 2:** Interobserver agreement for all nodules.

		between radiologists		between radiologist and CAD	
		meanKappa	meanSE	meanKappa	mean SE
100 mAs	120 kV	0.895	0.084	0.693	0.105
100 mAs	100 kV	0.908	0.087	0.641	0.113
100 mAs	80 kV	0.903	0.093	0.696	0.081
50 mAs	120 kV	0.881	0.069	0.528*	0.113
50 mAs	100 kV	0.853	0.073	0.481*	0.115
50 mAs	80 kV	0.880	0.070	0.518*	0.114
25 mAs	120 kV	0.898	0.068	0.592	0.125
25 mAs	100 kV	0.864	0.080	0.721	0.110
25 mAs	80 kV	0.801*	0.091	0.526*	0.127

Note - SE, standard error; mAs, Miliampèresecond; kVp, Kilovolt peek; CAD, computer assisted detection.

significant: p<0.05; compared to standard CT.

### Combined Sensitivities for the Radiologists and CAD

**Figure 2:**
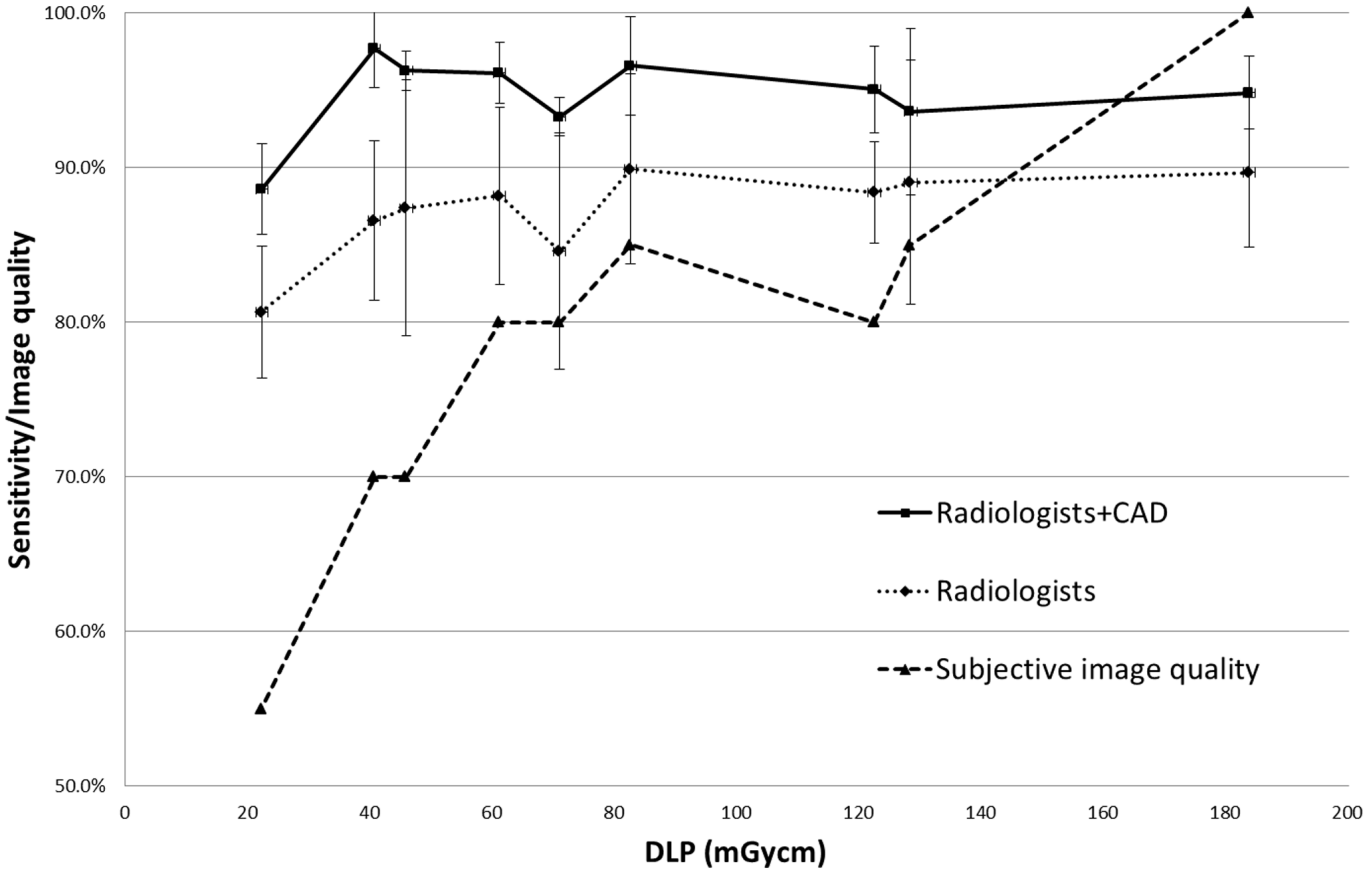
Dose dependent sensitivity for solid nodules and subjective image quality. The sensitivity of radiologists increased between 5 and 11% when CAD was used in combination with human assessment. When the dose was reduced, the subjective image quality dropped faster than the sensitivity, i.e., an accurate diagnosis could be made even when the subjective image quality was reduced.

At the standard dose level, the sensitivity for detecting solid nodules rose significantly by 5.1% to 94.8%±2.4% (p = 0.021) when the independent CAD data were combined with the radiologists’ data. When the dose was lowered, the additional sensitivity from CAD rose to a maximum of 11.1% for 25 mAs/100 kVp (Fig. 2). Individual and dose-dependent additions to the sensitivities varied from 0% to 16.7% (Appendix S1). There was no increase in the sensitivity of detecting GGNs on the standard CT, but at 100 mAs/100 kVp, the sensitivity increased by 4.2%±8.4% (p = 0.25). For all dose levels, the sensitivity for all nodules together rose significantly between 2.5% and 5.9% using CAD (p<0.05). At the standard dose, the sensitivity for all nodules increased significantly from 92.3% to 94.8%. With the exception of the 25 mAs/80 kVp dose, for which the sensitivity dropped significantly to 86.8%±3.0%, the combined sensitivity remained constant over all dose levels (Table 1).

### Nodule Detection Per Diameter and Per Nodule Type (Table 3)

The radiologists’ mean sensitivities for detecting solid nodules of 12, 10, 8 and 5 mm diameters at the standard CT dose were 90.2%±5.3%, 90.6%±8.8%, 95.1%±3.9% and 84.8%±10.5%, respectively. The differences in detectability were not significant (p>0.07). At the lowest dose level, the sensitivities dropped to 82.5%±10.5%, 89.1%±10.0%, 83.2%±9.7% and 66.1±10.9%, respectively; however, the decrease in sensitivity was only significant for the solid 5-mm nodule (p = 0.036). There were no other significant drops in sensitivity for any of the nodules when the dose was decreased from the standard level. The mean sensitivities of the radiologists for detecting GGNs of 12, 10, 8 and 5 mm diameter were 98.3%±1.0%, 98.4%±0%, 95%±2.7% and 86.0%±10.2%, respectively. These sensitivities decreased at the lowest dose to 87.4%±11.8%, 90.8%±8.4%, 90.8%±8.4% and 73.1%±17.1%, respectively; however, the decreases were not significant. The sensitivities of CAD as a standalone device were similar for all solid nodules except at the lowest dose level (Appendix S2). In contrast, when the dose was decreased for small GGNs, the sensitivity decreased significantly. For example, the CAD was not able to detect 5-mm GGNs at the lowest dose levels. For the smallest solid nodules (5 mm), when the data from the radiologists and the CAD were combined the sensitivity increased significantly by 9.9–25.3% at all dose levels. For all of the other nodules and dose levels, the sensitivity was not affected when the CAD reading was added.

### Subjective Image Quality

At the standard dose level, the median subjective score (on a scale from 1 to 5) was 5 ( = 100%). This value dropped significantly to 2.75 ( = 55%) at the lowest dose level (p<0.0001, Fig. 2).

### Longest Diameter and Volume Measurements of Solid Lung Nodules at the Standard and Reduced Doses

The mean longest diameter (according to the RECIST guidelines [28], [29]) of the 65 solid nodules at the standard CT dose was 8.6 mm. The absolute measurement error of the radiologist was an average of +0.10 mm±0.32 mm (SD), with an average relative measurement error of +1.5%±4.7%; the errors were not significant. The average volume of these nodules was 334.5 mm^3^. The radiologist overestimated the volume by an average of 3.8 mm^3^±31.8 mm^3^, with an average relative volume measurement error of 3.8%±11.7%; neither finding was significant. Similar results were achieved at all dose levels. The measurement error increased to +0.29 mm±0.39 mm at the lowest exposures; however, the error was not significant. The SD of the volume measurement differences at the standard CT dose (31.8 mm^3^) was significantly lower than the SD of the lowest dose CT (47.6 mm^3^), indicating a higher variance in the measurement differences at lower dose levels (p = 0.002). When the CAD was used as a semi-automated diameter and volume measurement tool, the measurement errors were +5.8%±8.7% and +13.7%±16.8%, respectively. The measurement errors and the SD were greater at lower dose levels; however, none of the errors was significantly different from the true volume (Table 3). The average volumetry errors of the radiologists and CAD at the lowest dose level (25 mAs/80 kVp) were −1.3%±10.4% and −8.0%±15.6%, respectively. In addition, at the second lowest dose level (25 mAs/100 kVp), there were no significant volumetry errors: −0.3%±7.9% and −1.3%±9.4% for the radiologists and CAD, respectively. The SD was highest at the lowest dose level: 9.2% and 15.6% for diameter and volume measurement differences, respectively. The volume measurement SD proved to be highly significant (<0.001), which means that there is a higher volatility in the measurement errors without a significant difference in the mean measurement. The per-diameter analysis at the standard dose level demonstrated non-significantly higher measurement errors for the radiologists in measuring the 5-mm nodules (9.3%±6.8%) compared to the other nodules (from −1% to +1.9%). These data were accompanied by a higher volume measurement error (Appendix S3). In absolute figures, the 5-mm nodules were overestimated by 0.5 mm±0.3 mm, which was not significant. At the lowest dose level, the measurement error of all nodules was between −1.3% and +5.3% for the diameter and between −9.8% and +6.7% for the volume; neither error was significant. In contrast, the CAD demonstrated significant measurement errors for larger nodules at higher dose levels (Table 4): the diameters of the 10- and 12-mm nodules were overestimated by 0.6 and 1.2 mm, corresponding to a volume measurement error between 36.6 and 60.2 mm^3^. There were no significant measurement errors for the 5- and 8-mm nodules or any nodules examined at the lower dose levels (25 and 50 mAs).

**Table 3:** Mean sensitivities per nodule diameter for each exposure level.

		GGN				SOLID			
Tube parameters		5 mm	8 mm	10 mm	12 mm	5 mm	8 mm	10 mm	12 mm
**Readers alone**									
100 mAs	120 kVp	86.0%±10.2%	95.0%±2.7%	98.3%±2.1%	98.3%±1.0%	84.8%±10.5%	95.1%±3.9%	90.6%±8.8%	90.2%±5.3%
100 mAs	100 kVp	85.3%±12.6%	97.5%±1.3%	91.0%±0.0%	97.5%±19.9%	84.2%±8.0%	88.2%±5.3%	90.8%±8.9%	91.0%±8.8%
100 mAs	80 kVp	75.4%±18.3%	88.8%±9.0%	90.4%±12.3%	90.4%±12.3%	78.5%±12.3%	80.9%±10.6%	91.3%±7.9%	91.3%±7.9%
50 mAs	120 kVp	84.8%±13.5%	98.9%±1.0%	98.9%±1.0%	91.3%±8.7%	79.2%±11.4%	91.9%±2.4%	94.2%±5.7%	92.1%±3.1%
50 mAs	100 kVp	80.1%±7.8%	95.8%±2.2%	93.1%±5.7%	91.3%±8.8%	83.4%±12.3%	88.5%±5.8%	93.1%±2.4%	90.8%±6.7%
50 mAs	80 kVp	72.3%±10.3%	94.4%±3.5%	92.5%±0.0%	87.1%±10.3%	74.4%±6.1%	95.0%±2.4%	92.8%±6.5%	92.9%±3.1%
25 mAs	120 kVp	85.1%±12.6%	98.8%±1.3%	92.8%±0.0%	95.2%±2.8%	75.0%±15.7%	90.8%±6.8%	92.5%±6.9%	92.5%±3.4%
25 mAs	100 kVp	75.7%±17.1%	91.9%±6.4%	93.9%±4.6%	93.9%±4.3%	73.8%±4.8%	91.5%±6.8%	91.2%±98.7%	93.5%±3.4%
25 mAs	80 kVp	73.1%±17.1%	90.8%±8.4%	90.8%±9.9%	87.4%±11.8%	66.1%±10.9%**	83.2%±9.7%	89.1%±10.0%	82.5%±10.8%
**Readers+CAD combined**									
**100 mAs**	**120 kVp**	**86.0%±10.2%**	**95.0%±4.5%**	**98.3%±1.0%**	**98.3%±0.9%**	**96.1%±2.3%**	**96.8%±1.1%**	**93.4%±4.3%**	**94.9%±3.9%**
100 mAs	100 kVp	88.7%±11.8%	99.6%±1.2%	94.1%±4.7%	99.6%±1.0%	94.6%±3.7%	92.6%±3.6%	92.0%±6.6%	93.3%±5.4%
100 mAs	80 kVp	77.8%±13.4%	92.8%±8.8%	93.2%±5.3%	93.2%±6.5%	95.0%±2.6%	89.4%±6.3%	96.3%±2.4%	96.3%±2.3%
50 mAs	120 kVp	84.8%±14.1%	98.9%±1.0%	98.9%±1.1%	91.3%±7.7%	91.8%±5.4%	97.5%±0.0%	97.5%±1.2%	97.4%±1.9%
50 mAs	100 kVp	82.1%±6.2%	97.8%±1.6%	93.6%±4.4%	91.8%±5.6%	93.3%±5.4%	96.1%±3.4%	96.1%±2.6%	96.8%±1.7%
50 mAs	80 kVp	74.1%±8.5%	94.6%±5.9%	92.8%±6.3%	87.4%±11.8%	93.1%±3.2%	98.8%±0.7%	96.5%±1.7%	96.6%±1.5%
25 mAs	120 kVp	85.3%±17.1%	99.1%±0.8%	96.0%±0.0%	95.4%±3.5%	91.0%±7.6%	95.2%±2.4%	97.0%±1.1%	99.3%±0.0%
25 mAs	100 kVp	76.0%±18.7%	94.4%±2.9%	94.4%±4.0%	94.4%±3.2%	99.1%±0.7%	97.8%±3.1%	95.3%±3.6%	97.6%±3.6%
25 mAs	80 kVp	73.1%±18.7%	90.8%±8.9%	90.8%±8.8%	87.4%±11.8%	77.1%±19.7%	91.4%±6.9%	95.5%±3.6%	88.5%±9.6%
**delta (combined-alone)**									
**100 mAs**	**120 kVp**	**0.0%±0.0%**	**0.0%±0.0%**	**0.0%±0.0%**	**0.0%±0.0%**	**11.3%±5.5%** *	**1.7%±1.0%**	**2.8%±4.8%**	**4.7%±3.5%**
100 mAs	100 kVp	3.5%±2.1%	2.1%±3.1%	3.1%±5.4%	2.1%±2.0%	10.3%±4.8%*	4.4%±2.3%	1.1%±2.1%	2.3%±2.4%
100 mAs	80 kVp	2.4%±1.9%	4.0%±4.5%	2.9%±6.5%	2.9%±6.0%	16.5%±6.8%*	8.5%±4.5%	5.0%±4.7%	5.0%±5.6%
50 mAs	120 kVp	0.0%±0.0%	0.0%±0.0%	0.0%±0.0%	0.0%±0.0%	12.6%±4.5%*	5.5%±3.4%	3.3%±2.5%	5.4%±4.3%
50 mAs	100 kVp	2.1%±2.4%	2.1%±2.2%	0.5%±0.9%	0.5%±0.5%	9.9%±3.7%*	7.6%±6.7%	3.1%±2.4%	6.0%±4.3%
50 mAs	80 kVp	1.8%±2.4%	0.3%±0.5%	0.3%±0.2%	0.2%±0.8%	18.7%±5.4%**	3.8%±4.0%	3.7%±2.9%	3.7%±2.6%
25 mAs	120 kVp	0.2%±0.6%	0.2%±0.7%	3.2%±3.0%	0.2%±0.3%	16.0%±6.6%*	4.4%±5.7%	4.4%±5.5%	6.8%±3.6%
25 mAs	100 kVp	0.4%±0.8%	2.5%±3.1%	0.4%±0.4%	0.4%±0.5%	25.3%±8.7%**	6.3%±4.6%	4.1%±5.5%	4.2%±4.5%
25 mAs	80 kVp	0.0%±0.0%	0.0%±0.0%	0.0%±0.0%	0.0%±0.0%	11.0%±5.1%	8.2%±4.5%	6.4%±3.6%	6.1%±3.6%

Note- GGN, gound glass nodule; mAs, Miliampèresecond; kVp, Kilovolt peek.

significant: p-value <0.05; compared to standard dose CT or to radiologists without CAD (delta).

significant: p-value <0.001; compared to standard dose CT or to radiologists without CAD (delta).

**Table 4:** RECIST-diameter- and volume-measuremet-errors of CAD for each nodule size and dose level.

		RECIST						VOLUME					
5 mm nodules		mean (mm)	sd	ME	sd	ME%	sd	mean (mm3)	sd	VME	sd	VME%	sd
100 mAs	120 kV	**5.3**	0.7	0.3	0.7	6.2%	14.2%	**75.3**	17.3	9.9	17.3	15.1%	26.5%
100 mAs	100 kV	**5.1**	0.4	0.1	0.4	1.3%	7.8%	**69.4**	13.6	4.0	13.6	6.1%	20.7%
100 mAs	80 kV	**5.1**	0.6	0.1	0.6	2.4%	11.8%	**73.1**	11.9	7.7	11.9	11.7%	18.2%
50 mAs	120 kV	**4.8**	0.4	−0.2	0.4	−3.9%	8.7%	**68.7**	10.1	3.2	10.1	4.9%	15.4%
50 mAs	100 kV	**4.9**	0.7	−0.1	0.7	−2.1%	14.4%	**67.8**	5.8	2.3	5.8	3.5%	8.8%
50 mAs	80 kV	**4.7**	0.3	−0.3	0.3	−6.8%	5.9%	**64.3**	7.0	−1.2	7.0	−1.8%	10.7%
25 mAs	120 kV	**4.8**	0.2	−0.2	0.2	−3.2%	3.5%	**71.5**	7.2	6.1	7.2	9.2%	11.1%
25 mAs	100 kV	**4.7**	0.3	−0.3	0.3	−5.8%	5.7%	**66.5**	6.6	1.1	6.6	1.6%	10.1%
25 mAs	80 kV	**4.7**	0.2	−0.3	0.2	−5.3%	4.9%	**64.2**	4.3	−1.3	4.3	−1.9%	6.6%
**8 mm nodules**		**mm**	**sd**	**ME**	**sd**	**ME%**	**sd**	**Vol**	**sd**	**VME**	**sd**	**VME%**	**sd**
100 mAs	120 kV	**8.3**	0.4	0.3	0.4	4.1%	4.7%	**277.8**	10.4	9.8	10.4	3.6%	3.9%
100 mAs	100 kV	**8.2**	0.3	0.2	0.3	3.0%	3.5%	**273.7**	10.3	5.6	10.3	2.1%	3.8%
100 mAs	80 kV	**8.3**	0.4	0.3	0.4	3.6%	4.8%	**278.5**	11.8	10.4	11.8	3.9%	4.4%
50 mAs	120 kV	**7.9**	0.2	−0.1	0.2	−1.1%	2.4%	**270.8**	11.4	2.7	11.4	1.0%	4.2%
50 mAs	100 kV	**7.9**	0.3	−0.1	0.3	−1.3%	4.2%	**273.2**	10.2	5.1	10.2	1.9%	3.8%
50 mAs	80 kV	**7.9**	0.2	−0.1	0.2	−1.5%	1.9%	**257.8**	10.6	−10.2	10.6	−3.8%	4.0%
25 mAs	120 kV	**7.9**	0.2	−0.1	0.2	−1.5%	3.1%	**271.2**	11.0	3.1	11.0	1.2%	4.1%
25 mAs	100 kV	**7.9**	0.4	−0.1	0.4	−1.0%	4.7%	**260.4**	20.5	−7.7	20.5	−2.9%	7.6%
25 mAs	80 kV	**7.6**	0.4	−0.4	0.4	−4.7%	4.9%	**227.4**	41.8	−40.7	41.8	−15.2%	15.6%
**10 mm nodules**		**mm**	**sd**	**ME**	**sd**	**ME%**	**sd**	**Vol**	**sd**	**VME**	**sd**	**VME%**	**sd**
100 mAs	120 kV	**10.6**	0.1	0.6*	0.1	5.8%*	1.3%	**570.8**	14.4	47.2*	14.4	9.0%*	2.7%
100 mAs	100 kV	**10.6**	0.3	0.6*	0.3	5.6%*	2.7%	**564.8**	16.5	41.2*	16.5	7.9%*	3.2%
100 mAs	80 kV	**10.5**	0.3	0.5*	0.3	5.4%*	2.6%	**560.2**	15.6	36.6*	15.6	7.0%*	3.0%
50 mAs	120 kV	**10.1**	0.3	0.1	0.3	1.2%	3.3%	**549.7**	15.9	26.1	15.9	5.0%	3.0%
50 mAs	100 kV	**9.9**	0.4	−0.1	0.4	−0.8%	3.6%	**539.9**	25.7	16.3	25.7	3.1%	4.9%
50 mAs	80 kV	**10.0**	0.2	0.0	3.1	−11.4%	31.3%	**520.9**	23.0	−2.7	65.1	−0.5%	31.5%
25 mAs	120 kV	**10.2**	0.7	0.2	0.7	1.6%	6.9%	**565.9**	151.0	42.3	151.0	8.1%	28.8%
25 mAs	100 kV	**10.3**	0.8	0.3	0.8	3.3%	7.8%	**555.8**	144.8	32.2	144.8	6.2%	27.7%
25 mAs	80 kV	**10.1**	0.7	0.1	0.7	1.1%	7.3%	**572.7**	151.1	49.1	151.1	9.4%	28.8%
**12 mm nodules**		**mm**	**sd**	**ME**	**sd**	**ME%**	**sd**	**Vol**	**sd**	**VME**	**sd**	**VME%**	**sd**
100 mAs	120 kV	**12.9**	0.7	0.9	0.7	7.5%	5.4%	**958.7**	32.8	53.9*	20.8	6.0%*	2.4%
100 mAs	100 kV	**13.0**	0.5	1.0	0.5	7.9%	4.5%	**966.3**	24.3	61.5*	24.3	6.8%*	2.7%
100 mAs	80 kV	**13.2**	0.5	1.2*	0.5	10%*	4.3%	**965.0**	21.0	60.2*	21.0	6.7%*	2.3%
50 mAs	120 kV	**12.0**	0.2	0.0	0.2	−0.2%	1.5%	**941.3**	19.1	36.5	19.1	4.0%	2.1%
50 mAs	100 kV	**12.1**	0.3	0.1	0.3	0.8%	2.5%	**904.5**	27.1	−0.3	27.1	0.0%	3.0%
50 mAs	80 kV	**12.0**	0.3	0.0	0.3	−0.4%	2.2%	**851.5**	113.1	−53.3	113.1	−5.9%	12.5%
25 mAs	120 kV	**12.3**	0.2	0.3	0.2	2.8%	1.7%	**924.8**	28.5	20.0	28.5	2.2%	3.2%
25 mAs	100 kV	**12.2**	0.2	0.2	0.2	1.3%	1.9%	**895.3**	9.5	−9.5	9.5	−1.1%	1.0%
25 mAs	80 kV	**12.2**	0.3	0.2	0.3	1.3%	2.2%	**876.6**	37.8	−28.2	37.8	−3.1%	4.2%

Note-ME: measurement error, sd: standard deviation, ME%: ME in percent of longest diameter, VME: volume measurement error,

VME%: VME in percent of nodule volume.

Significant measurement errors.

## Discussion

This study demonstrated the feasibility of a low-dose CT when both current and voltage are reduced together. The decrease in the combined sensitivity for detecting solid nodules was only significantly decreased at the lowest dose (25 mAs/80 kVp), which was primarily due to the significant decrease in sensitivity for detecting the solid 5-mm nodules at the lowest dose level. The sensitivity for detecting all other nodules did not decrease significantly at any dose level. The sensitivity threshold level of significance for the per diameter analysis was low because the number of nodules in each of the 8 categories was lower than in the combined analysis. Therefore, a dose reduction from 100 mAs/120 kVp to 25 mAs/100 kVp seems possible, equaling a reduction of the dose-length-product from 184 to 41 mGycm (a 78% dose reduction). At the second lowest dose level, no significant loss in sensitivity was demonstrated for any nodule size, nodule type, radiologist or combination of radiologist and CAD. These results are consistent with studies that focused on reducing either tube current or voltage separately [2], [3], [13], [35]. Rusinek et al. reported an acceptable tube current level of 20 mAs [35]. In previous studies, the advantage of reducing the CT tube voltage to 80 kVp was mainly assessed to increase contrast on CT angiography, especially to detect pulmonary embolisms [14]–[17]. Our results indicated that while a voltage level of 80 kVp was sufficient for detecting solid nodules, a minimum of 100 kVp was necessary to accurately detect GGNs. At all tube current levels, the sensitivity decreased when the voltage was 80 kVp. This finding supported the hypothesis that voltage has a greater impact on sensitivity than low tube current. These results suggest that the lowest CT tube parameters with acceptable image and diagnostic quality for cancer screening are 25 mAs and 100 kVp. However, as this was only a simulation, it will be necessary to conduct clinical studies to confirm this dose level.

A low kV theoretically leads to an increase in contrast, with a potentially improved detectability for GGNs; however, our data comparing the 25 mAs/100 kVp versus the 50 mAs/80 kVp dose levels did not support this theory. There is also doubt that this dose change might improve the sensitivity for solid nodules because the contrast is always very high (black and white) for lung nodule recognition in a lung window setting. The main difficulty encountered when detecting lung nodules is separating the nodules from the vessels, which is likely limited by the image noise. Interestingly, the sensitivity for detecting GGNs was higher than the sensitivity for detecting solid nodules at almost all dose levels. One likely reason for this difference is that there is an absence of areas with ground glass opacities in the phantom lung, which consisted of either black lung (air) or white lung (broncho-vascular tree and artificial solid nodules), therefore making it easier to detect structures with different attenuation, such as artificial GGNs.

When the dose level was decreased from the standard to the lowest dose, the subjective image quality dropped from 5 to 2.75, while the average sensitivity decreased by only 10%. This result confirmed the hypothesis that excellent image quality is not necessary for nodule or pattern detection [2]–[13].

The feasibility of detecting nodules with low-dose CAD was demonstrated in an earlier study [24]. In this study, CAD performed equally well at tube currents of 5 mAs and 75 mAs with a CT voltage level of 120 kVp. We additionally showed that CAD was able to detect solid nodules at a voltage level of 80 kVp. CAD was significantly inferior in detecting GGNs: CAD achieved a maximum sensitivity of only 59.1% at the standard CT dose compared to the 94.9% sensitivity achieved by the radiologists. Moreover, the CAD sensitivity for GGN detection dropped to 20.6% at the lowest dose. CAD demonstrated significant lower sensitivities for smaller GGNs at lower doses. This observation is in accordance with the manufacturer’s statement (MEDIAN) that the detection performance of the LMS CAD is optimized for solid pulmonary nodules. This result may present a serious problem in clinical detection because lung adenocarcinomas often appear as GGNs [36], [37]. Further studies are needed to investigate other CAD brands to confirm these results.

In our study, the radiologists’ capacity to detect solid nodules at the standard dose levels could be significantly improved by 5.1% using CAD. Previous investigators have also stated the usefulness of CAD [18]–[20], [38], with reports of between 5 and 20% increased sensitivity [19], [20]. In these studies, the radiologists’ sensitivity for solid nodule detection was closer to 60% than 100% without CAD, which meant that there was greater room for improvement when CAD was added. The impact of CAD on the radiologists’ sensitivity was even greater at lower dose levels. In our study, the sensitivity increased up to 11.1% at the lowest dose (25 mAs/100 kVp), which was likely due to the lower starting point of the sensitivity at lower dose levels. Because the capacity of CAD to detect GGNs was low, there was also a small impact on the sensitivity of detecting GGNs when CAD was combined with the radiologists’ findings (between 0% and 4.2%). Unexpectedly, the false-positive rates of the CAD decreased at lower dose levels and depicted mostly branching vessels.

Inter-observer variability among radiologists was greater than the agreement between CAD and the human eye; this difference demonstrated the capacity of the CAD to contribute additional accuracy in nodule detection. The best clinical scenario would be one in which high sensitivities for both the radiologists and CAD were combined with a poor agreement, which would lead to a maximum combined detection rate. The sensitivity of CAD divided by the K strength of agreement could be a new indicator for CAD accuracy. If CAD were used in place of a radiologist as the second reader, the greatest impact would be on the sensitivity of detecting solid nodules. Although the addition of CAD resulted in improved sensitivity, there were also more false positives, which negatively impacted the specificity. However, the use of CAD also helped to overcome the loss of image quality at lower dose levels. If the dose is lowered to 25 mAs/100 kVp, the addition of CAD can improve the sensitivity of solid nodule detection to 97.7%, which is higher than the sensitivity of human readers on standard CT without CAD (89.7%). It is important to note that these results are valid for phantoms and may not be applicable to a smoker’s lung. Clinical prospective studies are needed to address this issue.

We could not document a significant VME for any dose level in the combined nodule analysis for the radiologists or the semi-automated CAD. The VME for the radiologists was 3.8%±11.7%, which was lower than the 13.7%±16.8% VME for CAD. The overestimation of the nodule volume was not significant with either method. The per-nodule diameter analysis confirmed this finding for CAD and larger nodules. At higher dose levels, larger nodules were significantly overestimated. Xie et al. [25] reported volume underestimations of 26.4%± 15.5% and 7.6%± 8.5% for radiologists and semi-automated CAD, respectively. In addition, Willemink et al. reported volume underestimations ranging from –0.9% to –23.9% for a semi-automatic volume measurement software at comparable low-dose levels with nodules ≥5 mm [39]. In addition to the use of a different image reconstruction kernel and slice thickness, another plausible reason for this discordance may be differences in computers’ nodule border detection algorithms (segmentations). From the radiologist’s perspective, nodule measurement is likely experience-dependent. While beginners tend to include the full thickness of the ground glass transition zone around the nodule into the measurement, more experienced radiologists measure from the middle to the middle transmission zone. The variance in the volume measurement differences at the standard CT dose was significantly lower than the variance of the lowest CT dose, which indicates higher fluctuation and volatility of the measurement differences at lower dose levels without significant differences in the mean measurements for both the radiologists and the CAD.

### Limitations

This study is an anthropomorphic phantom study, and our results need to be confirmed in a clinical setting. In particular, the BMI of our phantom, which is based on an average Japanese male, is likely lower than the BMI of the average western white male; therefore, the investigated dose levels resulted in lower image noise levels, which may have positively influenced the sensitivity.

In addition, this study was conducted using a CT scanner equipped with filtered back-projection image reconstruction. Further research is likely necessary to find the lowest dose levels with iterative image reconstruction in lung nodule detection.

We did not investigate the dependency of nodule location on sensitivity; however, the high number and random placement of nodules permitted a realistic simulation. Another limitation is that only spherical lesions were used, which is certainly not the typical shape of a malignant nodule; therefore, it may have been more difficult to detect round nodules surrounded by round-to-ovoid vessels. Irregular lesions may be prone to higher variances/measurement errors as scan parameters are changed. In addition, the HU value of the solid nodules was higher (100 HU) than most ‘human’ solid nodules. Moreover, the density of ‘lung tissue’ in the phantom is below normal, as there is no tissue in the phantom. This factor may lead to an overestimation of the sensitivity.

We did not focus on specificity in our study setting. A true negative would be defined as a negative phantom; however, the study was designed for nodules and not for phantoms. Furthermore, although specificity is important, it is secondary in a study that primarily addresses screening.

## Conclusion

Radiologists’ sensitivity for detecting solid lung nodules (86–90%) was only impaired when the tube current and voltage were both decreased to 25 mAs/80 kVp (81%). Independent of the tube current, the sensitivity for detecting GGNs was significantly lower at all dose levels at 80 kVp. The CAD and the radiologists had similar sensitivities for detecting solid nodules; however, the CAD had a lower sensitivity for detecting GGNs when used as a standalone device. The CAD significantly increased the radiologists’ sensitivity for detecting solid nodules at all dose levels (5–11%). Based on our results, a low-dose CT protocol using 25 mAs and 100 kVp with conventional filtered back-projection image reconstruction is an acceptable method for detecting nodules in an anthropomorphic lung phantom.

## Supporting Information

Appendix S1 **Sensitivities of individual readers with and without CAD for each exposure level.**
(XLSX)

Appendix S2 **CAD sensitivities per nodule diameter for each exposure level.**
(XLSX)

Appendix S3 **RECIST-Diameter- and Volume-measurement-errors of radiologist for each nodule size.**
(XLSX)
